# Maternal and newborn outcomes in Pakistan compared to other low and middle income countries in the Global Network’s Maternal Newborn Health Registry: an active, community-based, pregnancy surveillance mechanism

**DOI:** 10.1186/1742-4755-12-S2-S15

**Published:** 2015-06-08

**Authors:** Omrana Pasha, Sarah Saleem, Sumera Ali, Shivaprasad S Goudar, Ana Garces, Fabian Esamai, Archana Patel, Elwyn Chomba, Fernando Althabe, Janet L Moore, Margo Harrison, Mabel B Berrueta, K Michael Hambidge, Nancy F Krebs, Patricia L Hibberd, Waldemar A Carlo, Bhala Kodkany, Richard J Derman, Edward A Liechty, Marion Koso-Thomas, Elizabeth M McClure, Robert L Goldenberg

**Affiliations:** 1 Department of Community Health Sciences, Aga Khan University, Karachi, Pakistan; 2 KLE University’s Jawaharlal Nehru Medical College, Belgaum, India; 3 Fundación para la Alimentación y Nutrición de Centro América y Panamá (FANCAP), Guatemala City, Guatemala; 4 Moi University School of Medicine, Eldoret, Kenya; 5 Indira Gandhi Government Medical College and Lata Medical Research Foundation; 6 University Teaching Hospital, University of Zambia, Lusaka, Zambia; 7 Institute for Clinical Effectiveness and Health Policy, Buenos Aires, Argentina; 8 RTI International, Durham, NC, USA; 9 Department of Obstetrics and Gynecology, Columbia University, New York, NY, USA; 10 University of Colorado School of Medicine, Denver, CO, USA; 11 Massachusetts General Hospital for Children, Boston, MA, USA; 12 University of Alabama at Birmingham, Birmingham, AL, USA; 13 Christiana Care Health Services, Newark, DE, USA; 14 Indiana University School of Medicine, Indianapolis, IN, USA; 15 Eunice Kennedy Shriver National Institute of Child Health and Human Development, Bethesda, MD, USA

**Keywords:** Pakistan, maternal mortality, stillbirth, neonatal mortality

## Abstract

**Background:**

Despite global improvements in maternal and newborn health (MNH), maternal, fetal and newborn mortality rates in Pakistan remain stagnant. Using data from the Global Network’s Maternal Newborn Health Registry (MNHR) the objective of this study is to compare the rates of maternal mortality, stillbirth and newborn mortality and levels of putative risk factors between the Pakistani site and those in other countries.

**Methods:**

Using data collected through a multi-site, prospective, ongoing, active surveillance system to track pregnancies and births in communities in discrete geographical areas in seven sites across six countries including Pakistan, India, Kenya, Zambia, Guatemala and Argentina from 2010 to 2013, the study compared MNH outcomes and risk factors. The MNHR captures more than 60,000 deliveries annually across all sites with over 10,000 of them in Thatta, Pakistan.

**Results:**

The Pakistan site had a maternal mortality ratio almost three times that of the other sites (313/100,000 vs 116/100,000). Stillbirth (56.5 vs 22.9/1000 births), neonatal mortality (50.0 vs 20.7/1000 livebirths) and perinatal mortality rates (95.2/1000 vs 39.0/1000 births) in Thatta, Pakistan were more than twice those of the other sites. The Pakistani site is the only one in the Global Network where maternal mortality increased (from 231/100,000 to 353/100,000) over the study period and fetal and neonatal outcomes remained stagnant. The Pakistan site lags behind other sites in maternal education, high parity, and appropriate antenatal and postnatal care. However, facility delivery and skilled birth attendance rates were less prominently different between the Pakistani site and other sites, with the exception of India. The difference in the fetal and neonatal outcomes between the Pakistani site and the other sites was most pronounced amongst normal birth weight babies.

**Conclusions:**

The increase in maternal mortality and the stagnation of fetal and neonatal outcomes from 2010 to 2013 indicates that current levels of antenatal and newborn care interventions in Thatta, Pakistan are insufficient to protect against poor maternal and neonatal outcomes. Delivery care in the Pakistani site, while appearing quantitatively equivalent to the care in sites in Africa, is less effective in saving the lives of women and their newborns. By the metrics available from this study, the quality of obstetric and neonatal care in the site in Pakistan is poor.

**Trial registration:**

The study is registered at clinicaltrials.gov [NCT01073475].

## Background

The time around delivery and the postnatal period is the most vulnerable for both mother and newborn. Annually almost 300,000 maternal deaths [1], over two and half million stillbirths [2] and a similar number of neonatal deaths [3] occur worldwide, the majority occurring during delivery and in the early postpartum period. Over the last 25 years there have been substantial improvements in maternal and newborn outcomes in many countries [4]. However, the progress has been uneven, drawing the inequities in maternal and neonatal health between and within countries into sharper focus [5]. While the preponderance of these deaths occur in South Asia and sub-Saharan Africa [6], there are countries such as Rwanda [7] that have made substantive strides in reducing both maternal and neonatal mortality. Unfortunately, other countries have been less successful. In fact, about a quarter of countries with the poorest outcomes have made insufficient progress or none at all [8].

In South Asia, Pakistan is one of the countries where maternal mortality ratios (MMR) and neonatal mortality rates remain stagnant. The Pakistan Demographic and Health Survey (PDHS) 2012-13 reports a perinatal mortality rate of 75 per 1000 pregnancies and neonatal mortality rate of 55 per 1,000 live births [9]. The neonatal mortality rate was not substantially different between PDHS 1990-91 and PDHS 2012-13. Over the same period there was a 19% reduction in infant mortality and 24% reduction in under-5 mortality in Pakistan [9]. While progress towards the health-related Millennium Development Goals has been limited in Pakistan overall, the inability to provide safe delivery and postpartum care are the most glaringly inadequate.

A recent report on early neonatal mortality and stillbirth estimates that a child being delivered in Pakistan has the highest risk in the world of both intrapartum stillbirth and death on the day of birth [10]. In comparison to its neighbors as well as developing countries with a similar level of economic development and international investment in MNH worldwide, Pakistan has had remarkably limited success in reducing maternal and newborn deaths [11]. In many of the countries with similarly poor peripartum outcomes, there are clear explanatory factors for poor maternal and newborn outcomes. For example, Sierra Leone, Somalia and Guinea-Bissau experience grinding poverty, which likely explains poor outcomes in these countries. In comparison, Pakistan has a gross national income per capita at least twice as high, yet still has MNH outcomes worse than all the aforementioned countries in sub-Saharan Africa. HIV infection significantly undermines maternal newborn health and may contribute to poor outcomes [12], but the HIV infection rate amongst Pakistani women presenting for antenatal care remains remarkably low, so cannot account for poor outcomes in Pakistan [13]. Even war-torn, neighboring Afghanistan has made greater strides in improving MNH outcomes than Pakistan [14].

Attempts to explain the failure of Pakistan to improve maternal and newborn health have focused on the situation in-country. At the macro level, the lack of female education, a high rate of population growth, a lack of integration between vertical healthcare programs with overlapping objectives, poor penetration of known life-saving healthcare interventions, low birth weight and poverty have been highlighted as possible reasons for poor MNH outcomes [11,15].

The Global Network for Women’s and Children’s Health Research’s Maternal Newborn Health Registry (MNHR) [16] provides a unique opportunity to compare MNH outcomes and putative explanatory factors at a surveillance site in Pakistan with sites in six other countries with comparable economic and developmental indicators. The purpose of this paper is to compare the rate of maternal mortality, stillbirth and newborn mortality between the site in Thatta, Pakistan and six other sites in the Registry, including two sites in India, as well as sites in Kenya, Zambia, Argentina and Guatemala.

## Methods

We used data collected by the *Eunice Kennedy Shriver* National Institute of Child Health and Human Development (NICHD)’s Global Network for Women and Children’s Health Research, a multi-site, prospective, ongoing, active surveillance system to track pregnancies and births in 100 clusters in Corrientes, Argentina; Chimaltenango, Guatemala; Nagpur District and Karnataka District, India; western Kenya; Thatta District, Pakistan; and Lusaka, Zambia [16]. These sites were selected by the NICHD to represent rural or semi-urban geographical areas in low to upper-middle income countries. The number of clusters varies from a minimum of six in Corrientes, Argentina to twenty-four in Belgaum, Karnataka, India, including twenty in Thatta, Pakistan.

The study site(s) in each country are described in detail elsewhere [17]. Thatta, the site in Pakistan, is a predominantly rural district bordering the two largest cities in the province of Sindh, Karachi and Hyderabad. Despite its close proximity to these urban centers, in 2003, Thatta was ranked 64^th^ of 91 districts in the country on the Human Development Index [18]. More recent reports show the education sector, in particular, lags behind the rest of the country. Thatta has the lowest educational attainment score in the province and is ranked amongst the 5 lowest in the country [19]. On the other hand, Thatta does have a large number of health care providers, spread throughout the district and the proportion of women delivering at health care facilities is higher than the national average [20].

Through the MNHR, all pregnant women who are residents of the study clusters are recruited, enrolled, and followed to delivery and the post-partum period. Information about the health of the mother and infant during the antenatal, labor and delivery and postnatal period is collected. Each cluster has a minimum of 300 deliveries per year and data from all consenting pregnant women are included in the MNHR database. The MNHR is described in detail elsewhere [16].

In brief, information on the eligible pregnant women and their babies is obtained at three time points. The first visit, at enrollment, ideally occurs by week 20 of gestation and information on the date of last menstrual period, estimated delivery date, age, level of schooling, parity, and status of last child born is collected. The second visit occurs within 48 hours of delivery and information collected includes prenatal care, birth preparedness, complications occurring during pregnancy, details of labor and delivery, including place, mode of delivery, provider and practices birth weight, status of the mother and newborn following delivery, referrals, and treatment provided to the mother and newborn at referral facilities. Interval maternal and newborn health and vital status is assessed at a third visit on day 42 after birth. The same study protocol and similar operational mechanisms are implemented at all the sites across the Network.

The study has been reviewed and approved at all of the involved institutions’ ethics review committees including the committees in the US institutions that partnered with each of the foreign sites. A Data Monitoring Committee appointed by NICHD reviews the MNHR data on an annual basis. All women provide consent to participate in the MNHR study.

## Analysis

We compared the data collected in Kenya, Zambia, Argentina, Guatemala and the two sites in India with that from Pakistan for the years 2010-2013. Results from the sites are grouped into those from India, from Africa and from Latin America. Data were entered at each study site, where data edits were performed prior to transmission to a central data center (RTI International, Durham, NC) where additional data edits were performed.

Data were analyzed centrally and statistical analyses performed using SAS v. 9.3. Descriptive analyses are reported for the delivery and health care characteristics, stratified by region. We modeled the risk of maternal mortality, stillbirth, and neonatal mortality and calculated point and interval estimates of risk ratios using multivariable generalized linear regression models; we used generalized estimating equations to account for correlation of outcomes within clusters to assure appropriately sized p-values and confidence intervals. To evaluate changes in outcomes over times, we modelled year of delivery and tested for trends across time with an orthogonal polynomial linear contrast.

## Results

Over the four years (2010-2013), data on a total of 48,273 pregnancies in Pakistan and 221,437 pregnancies in the six other sites were collected. Across all sites, among those enrolled, follow-up at 6 weeks after delivery was 98.4% (ranging from 97.5% to 99.9%). In this sample, the Pakistani site had a maternal mortality ratio (MMR) almost three times that of the sites. Similarly, the stillbirth, neonatal mortality and perinatal mortality rates in Pakistan were more than twice that of the other sites (Table 1).

**Table 1:** Maternal, fetal and neonatal mortality rates (2010-2013) in the NICHD Global Network’s Maternal Newborn Health Registry sites, comparing Thatta, Pakistan with sites in India, Africa and Latin America

	Pakistan	India	Africa	Latin America	Total except Pakistan
Sites	Thatta, Sindh	Nagpur, Maharashtra Belgaum, Karnataka	Kafue/Chongwe, Zambia Western Province, Kenya	Chimaltenango, Guatemala Corrientes, Argentina	

Births, N	48,868	119,785	63,976	39,557	223,318

Clusters, N	20	40	30	22	

42-day maternal mortality ratio, n (rate/100,000 LB)	144 (313)	142 (122)	76 (121)	35 (90)	253 (116)

Stillbirth, n (Rate/1,000)	2,760 (56.5)	3,068 (25.6)	1,356 (21.2)	681 (17.2)	5,105 (22.9)

Perinatal mortality, n (Rate/1,000)	4,589 (95.2)	5,303 (44.3)	2,188 (34.4)	1,197 (30.4)	8,688 (39.0)

28-d Neonatal mortality, n (Rate/1,000)	2,270 (50.0)	2,755 (23.6)	1,020 (16.4)	719 (18.6)	4,494 (20.7)

All of the sites in the Network except Pakistan have seen a reduction of one third or more in the adjusted MMR over the study period. The MMR in Thatta, Pakistan rose more than 50% from 219 per 100,000 live births in 2010 to 333 in 2013. This increase in the MMR represents a statistically significant trend over the study period (p=0.0325). At the other sites there has been a declining trend with the reduction in MMR at the Indian sites reaching statistical significance (p=0.0409) (Table 2).

**Table 2:** Maternal mortality ratios and stillbirth, neonatal and perinatal rates and the percent change 2010 and 2013, for Thatta, Pakistan and the Indian, African and Latin American sites

	Pakistan	India	Africa	Latin America
Sites	Thatta, Sindh	Nagpur, Maharashtra Belgaum, Karnataka	Kafue/Chongwe, Zambia Western Province, Kenya	Chimaltenango, Guatemala Corrientes, Argentina

42-day maternal mortality ratio/100,000 deliveries				

2010 adjusted risk estimate [95% CI]	219 (159, 302)	153 (117, 200)	143 (95, 216)	136 (75, 244)

2013 adjusted risk estimate [95% CI]	333 (233, 476)	98 (66, 146)	88 (56, 138)	70 (31, 154)

Change 2010 to 2013 (%)	54.3% increase	35.9% decrease	38.5% decrease	48.5% decrease

P-value for 2010-2013 trend test	0.0325	0.0409	0.1825	0.1724

Stillbirth, rate/1,000 births				

2010 adjusted risk estimate (95% CI)	55.1 (49.5, 61.3)	30.0 (27.4, 32.9)	23.7 [19.6, 28.6]	19.2 (15.9, 23.1)

2013 adjusted risk estimate (95% CI)	58.3 (51.0, 66.6)	23.4 (21.5, 25.3)	20.6 [16.8, 25.4]	15.4 (12.8, 18.4)

Change 2010 to 2013 (%)	5.8% increase	22.0% decrease	13.1% decrease	19.8% decrease

P-value for 2010-2013 trend test	0.7550	0.0003	0.7192	0.0489

28-d Neonatal mortality, rate/1,000 live births				

2010 adjusted risk estimate (95% CI)	48.5 (42.3, 55.7)	25.5 (23.1, 28.2)	20.7 (16.7, 25.7)	19.5 (14.7, 25.9)

2013 adjusted risk estimate (95% CI)	46.7 (40.9, 53.4)	25.6 (23.6, 27.7)	13.9 (11.6, 16.6)	18.7 (16.1, 21.7)

Change 2010 to 2013 (%)	3.7% decrease	0.4% increase	32.9% decrease	4.1% decrease

P-value for 2010-2013 trend test	0.2609	0.5975	0.0449	0.3984

Perinatal mortality, rate/1,000 births				

2010 adjusted risk estimate (95% CI)	91.6 (83.1, 100.8)	49.8 (45.9, 54.1)	39.7 (34.0, 46.5)	34.1 (28.9, 40.3)

2013 adjusted risk estimate (95% CI)	93.2 (83.8, 103.7)	43.4 (40.9, 46.0)	32.3 (27.0, 38.6)	28.0 (24.6, 31.9)

Change 2010 to 2013 (%)	1.7% increase	12.9% decrease	18.6% decrease	17.9% decrease

P-value for 2010-2013 trend test	0.5501	0.0089	0.3437	0.0050

*The mortality risk estimates were adjusted for study cluster.

The stillbirth, perinatal mortality and neonatal mortality rates remained essentially unchanged in Pakistan between 2010 and 2013. In contrast, each of the other sites had significant reductions in at least one indicator related to the fetus/newborn. In the Indian sites, there was a reduction of over 20% in the stillbirth rate, with a significant trend (p=0.0003), whereas in the African sites 28-day neonatal mortality fell by one-third (p=0.045). In the sites in Latin America, both the stillbirth rate (p=0.049) and the perinatal mortality rate (p=0.005) decreased by almost 20% each. Thus, adverse newborn outcomes in Pakistan were twice as high as any of the other sites in 2010 and the improvements at the other sites over the study period increased that difference to almost three times in 2013 (Table 2).

Reviewing maternal characteristics, which could explain the large differences in maternal outcomes between the Pakistan site and the sites across India, Africa and in Latin America, education and parity were notably different in the Pakistan site. More than 80% of the pregnant women in the Pakistani site had no formal education compared to a combined rate of 12.5% across the other sites. Women in the Pakistani site were more likely to have a higher parity than any of the other sites. More than a quarter of the women had a parity of more than 4 at the time of the included pregnancy and mean parity was twice that of the other sites. Maternal age and body mass index (BMI) were not substantially different between the Pakistani and the other sites. In fact, the BMI of Pakistani and Indian women was similar to each other, whereas, the BMI for women at the sites in Africa and Latin America was higher than the Asian sites. We examined the mean height of women in the different sites. Pakistani women were about 3 cm taller than the women in the Indian and Latin American sites, but shorter than the women in the African sites. Another explanatory factor for poor maternal and neonatal outcomes may be the dearth of antenatal care for women in Pakistan. They are less likely to have at least three antenatal care visits, immunization with tetanus toxoid and prenatal iron/vitamin supplementation than women at any of the other sites (Table 3).

**Table 3:** Maternal characteristics and antenatal care provision (2010-2013) in the NICHD Global Network’s Maternal Newborn Health Registry site in Thatta, Pakistan compared with sites in India, Africa and Latin America

	Pakistan	India	Africa	Latin America	Total except Pakistan
Sites	Thatta, Sindh	Nagpur, Maharashtra Belgaum, Karnataka	Kafue/Chongwe, Zambia Western Province, Kenya	Chimaltenango, Guatemala Corrientes, Argentina	

Deliveries, N*	48,273	118,889	63,259	39,289	221,437

Maternal age, N (%)					

< 20	1,854 (3.9)	8,389 (7.1)	14,745 (23.3)	7,479 (19.1)	30,613 (13.8)

20-35	43,563 (90.6)	110,149 (92.7)	44,725 (70.8)	27,891 (71.1)	182,765 (82.6)

> 35	2,682 (5.6)	245 (0.2)	3,684 (5.8)	3,867 (9.9)	7,796 (3.5)

Maternal education, N (%)					

No formal education	39,972 (83.2)	17,596 (14.9)	3,960 (6.3)	5,954 (15.2)	27,510 (12.5)

Primary	3,651 (7.6)	33,058 (28.0)	40,498 (64.3)	24,659 (63.0)	98,215 (44.6)

Secondary	2,818 (5.9)	52,423 (44.3)	16,787 (26.6)	8,102 (20.7)	77,312 (35.1)

University or higher	1,629 (3.4)	15,150 (12.8)	1,783 (2.8)	444 (1.1)	17,377 (7.9)

Parity, N (%)					

0	10,027 (20.8)	52,708 (44.5)	16,401 (26.0)	11,417 (29.1)	80,526 (36.5)

1-2	15,409 (32.0)	59,356 (50.1)	24,088 (38.1)	14,336 (36.6)	97,780 (44.3)

3-4	10,535 (21.9)	5,805 (4.9)	14,105 (22.3)	6,723 (17.1)	26,633 (12.1)

> 4	12,171 (25.3)	516 (0.4)	8,582 (13.6)	6,736 (17.2)	15,834 (7.2)

Parity, Mean (n, std)	3.0 (48,142, 2.8)	0.8 (118,385, 0.9)	2.1 (63,176, 2.0)	2.3 (39,212, 2.5)	1.5 (220,773, 1.8)

BMI**, Mean (n, std)	21.0 (47,992, 3.6)	20.0 (113,192, 2.8)	23.3 (27,476, 3.3)	26.5 (19,541, 4.2)	21.4 (160,209, 3.9)

Maternal height, cm, Mean (n, std)	154.5 (48024, 5.7)	151.8 (113574, 5.5)	158.4 (27544, 6.4)	150.8 (25473, 7.9)	152.7 (166591, 6.6)

ANC visits, N (%)					

0	3,139 (11.2)	32 (0.1)	357 (1.1)	463 (1.9)	852 (0.7)

1-2	12,946 (46.0)	4,902 (8.0)	6,879 (21.7)	2,491 (10.3)	14,272 (12.2)

≥ 3	12,064 (42.9)	56,174 (91.9)	24,393 (77.1)	21,316 (87.8)	101,883 (87.1)

Tetanus toxoid vaccine, N (%)	25,074 (52.0)	118,561 (99.8)	58,503 (92.5)	28,436 (72.9)	205,500 (93.0)

Vitamins/Iron, N (%)	31,533 (65.4)	118,087 (99.5)	60,159 (95.1)	35,152 (89.9)	213,398 (96.6)

*Numbers less than the total reflect missing data **BMI data unavailable for Kenya site

With regard to delivery care, the Pakistani site differs significantly from the Indian sites but not as much from the other sites. In the Pakistani site, approximately 45% of deliveries take place at home or a location outside a health care facility. In the Indian sites, this number is less than 6%. On the other hand, in the Latin American sites, 42% of deliveries occur outside a health care facility and in Africa about half occur outside a health facility. A similar variation occurs for delivery attendants: in the Indian sites only 5% of deliveries are conducted by non-skilled personnel, whereas in the Latin American sites 41%, in the Pakistani site, 48% and in the African sites 51% of the deliveries are conducted by non-skilled personnel. Likewise, the Pakistan site is not significantly different in terms of deliveries that end in caesarean section. The percentage of operative deliveries range from 1.3% in the African sites to 22.8% in the Latin American sites: The Pakistani (9.4%) and Indian sites (15.9%) fall between these rates (Table 4).

**Table 4:** Delivery care (2010-2013) in the NICHD Global Network’s Maternal Newborn Health Registry site in Thatta, Pakistan compared with sites in India, Africa and Latin America

	Pakistan	India	Africa	Latin America	Total except Pakistan
Sites	Thatta, Sind	Nagpur, Maharashtra Belgaum, Karnataka	Kafue/Chongwe, Zambia Western Province, Kenya	Chimaltenango, Guatemala Corrientes, Argentina	

Deliveries, N	48,273	118,889	63,259	39,289	221,437

Delivery location, N (%)					

Hospital	13,913 (28.9)	80,472 (67.7)	8,048 (12.7)	21,605 (55.0)	110,125 (49.8)

Clinic	11,986 (24.9)	31,440 (26.5)	23,569 (37.3)	1,350 (3.4)	56,359 (25.5)

Home/Other	22,313 (46.3)	6,889 (5.8)	31,638 (50.0)	16,328 (41.6)	54,855 (24.8)

Birth attendant, N (%)					

Physician	12,355 (25.6)	70,260 (59.1)	1,343 (2.1)	19,865 (50.6)	91,468 (41.3)

Nurse/Midwife/HW	12,822 (26.6)	42,441 (35.7)	29,829 (47.2)	3,204 (8.2)	75,474 (34.1)

TBA	21,742 (45.1)	3,058 (2.6)	23,111 (36.5)	16,069 (40.9)	42,238 (19.1)

Family/Other	1,306 (2.7)	3,083 (2.6)	8,973 (14.2)	142 (0.4)	12,198 (5.5)

Delivery mode, N (%)					

Vaginal	40,671 (85.1)	99,041 (83.4)	61,657 (97.5)	30,293 (77.1)	190,991 (86.3)

Vaginal assisted	2,664 (5.6)	741 (0.6)	739 (1.2)	39 (0.1)	1,519 (0.7)

C-section	4,485 (9.4)	18,912 (15.9)	853 (1.3)	8,949 (22.8)	28,714 (13.0)

BA Gloves, N (%)					

Yes	36,922 (76.7)	114,844 (97.4)	61,988 (98.4)	38,511 (98.9)	215,343 (97.9)

Clean razor, N (%)					

Yes	47,107 (97.9)	116,381 (99.3)	62,386 (99.0)	26,818 (72.9)	205,585 (94.7)

Fetal heart rate taken, N (%)	48,221	118,838	63,256	39,284	221,378

Yes	20,444 (42.4)	114,743 (96.6)	40,380 (63.8)	28,339 (72.1)	183,462 (82.9)

Placed skin-to-skin after birth, N (%)					

Yes	966 (2.0)	34,320 (29.0)	15,152 (24.0)	14,451 (37.1)	63,923 (29.0)

Medicinal cord care, N (%)	48,381	118,636	63,240	39,045	220,921

Yes	2,978 (6.2)	44,579 (37.6)	19,967 (31.6)	24,734 (63.3)	89,280 (40.4)

Immunization, N (%)	48,379	118,010	63,240	38,800	220,050

Yes	698 (1.4)	30,025 (25.4)	7,034 (11.1)	17,769 (45.8)	54,828 (24.9)

Breast feeding within 1 hour, N (%)					

Yes	10,726 (22.1)	96,896 (83.4)	53,858 (84.5)	30,629 (77.8)	181,383 (82.7)

Simple indicators for quality of delivery care show that while Pakistani women may be as likely to have a facility-based delivery and skilled birth attendance as women in the Latin American sites or the African sites, the quality of that care is poorer. In the Pakistani site, only three-quarters of delivery attendants used gloves during the delivery compared to more than 97% in all other sites. Similarly, the fetal heart rate was measured in less than half of the deliveries in the Pakistani site, compared to more than 80% in the other sites. Postnatal care is substantially worse in the Pakistani site than in the other sites as evidenced by much lower rates of skin-to-skin (2.0% vs. 29.0%), medicinal cord care (6.2% vs. 40.4%), immunization (1.4% vs. 24.9%), and early initiation of breast-feeding (22.1% vs. 82.7%), with the latter percentages representing the mean rates of the other sites, combined.

**Figure 1:**
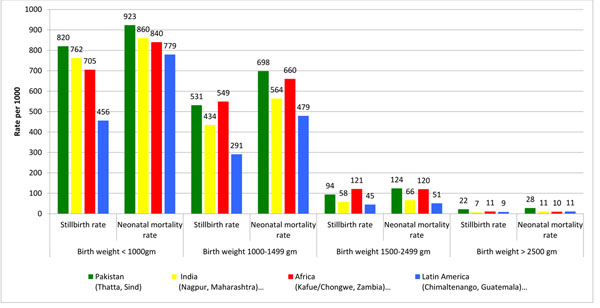
Stillbirth and Neonatal mortality rates by birth weight (2010-2013) in the NICHD Global Network’s Maternal Newborn Health Registry site in Thatta, Pakistan compared with sites in India, Africa and Latin America

The Pakistani site has the highest proportion of children born with a birth weight below 2500 g (17.9%). In comparison, the proportion of low birth weight (LBW) babies is 15.5% in the Indian sites, 11.5% in Latin America and only 4.7% in the African sites. In looking at mortality rates amongst extremely low birth weight babies and very low birth weight babies, in both the Pakistani and the African sites, approximately one in ten fetuses/neonates weighing less than 1500 g survive. At the Indian and Latin American sites, almost twice or three times as many newborns survive. Amongst babies with a birth weight between 1500-2499 g, the situation is similar, with babies in both Pakistan and the African sites at equally high risk of a poor outcome and babies at the Indian and Latin American sites faring better. The difference in mortality rates between the Pakistani site and the other sites is accounted for by the babies that are born with a birth weight ≥2500 g with the Pakistani site having mortality outcomes in infants weighing ≥2500 g at least twice as high as any of the other sites (Figure 1).

## Discussion

The MNHR is a network of surveillance sites, which provide detailed data in seven specific geographic sites in six countries across three continents. In the absence of well-functioning vital registration mechanisms in these countries, such demographic surveillance sites can provide detailed information not otherwise available. It is particularly useful for comparisons between the sites as data gathering mechanisms are similar, applying standard definitions across the sites in the three continents.

The MNHR demonstrates the large and growing cross-country disparity in newborn and particularly maternal outcomes from 2010 to 2013. The site in Pakistan fares worse than those in neighboring India and also lags well behind the sites in both Latin America and Africa. These data confirm the findings of recent national demographic and health surveys in these countries and other estimates for maternal mortality, stillbirth rates as well as perinatal and neonatal mortality. There is also a lack of improvement in newborn health outcomes in the Pakistani site over the study period, which is also reflected in the stagnating rates over interval measurements by serial national level surveys [9].

Particularly concerning is the increase in the measured maternal mortality ratio over the study period in Thatta, Pakistan. Even though maternal mortality is a relatively rare outcome, the MNHR captured a statistically significant increasing trend in the MMR in the Pakistani site. PDHS 2006-07 provided the last population-based MMR estimate in the country (276/100,000 live births) [10]. Subsequently, a projection of 260 was made in 2010 using a statistical model [21]. Most recently, an MMR of 299 was estimated using facility-based data from 2011 [22]. The burden of disease analysis projects an MMR of 400/100,000 live births in Pakistan in 2013 [1]. The MNHR, which identifies maternal deaths prospectively amongst an existing cohort of pregnant women in the catchment area has maternal mortality ratios that correlate closely with the most recent Burden of Disease Project estimates [1].

Amongst the underlying differences that may explain the disparate MNH outcomes across the sites, one of the starkest differences between Pakistan and the other sites is in maternal education. Nisar et al have described a significant increase in the risk of neonatal mortality associated with a lack of parental education in Pakistan during the 2006-07 PDHS [15]. Similarly, the findings of the WHO Multi-country Survey on Maternal and Newborn Health in Pakistan demonstrate the increased risk of poor maternal outcome related to lack of education [22]. Given that vast majority of women in the Pakistani site lack any formal education, the impact of being uneducated is likely to be considerable on both the mother and the child.

Another maternal characteristic that may affect both maternal and newborn outcomes and is significantly different between women at the Pakistani site and the others is the average total parity of each woman. On average, the parity of women at the site in Pakistan was twice that of any of the other sites. The most notable difference was with the Indian sites, where almost half of all pregnant women were nulliparous. The African and Latin American sites had higher average parity than the Indian sites; however, Pakistani women in Thatta were almost twice as frequently grand multiparous than women in the other sites. While a relationship between poor pregnancy outcome and grand multiparity has not been previously demonstrated in Pakistan, there is substantive evidence from other developing countries that high parity and reduced inter-pregnancy interval increase the risk of poor maternal and perinatal outcomes [23,24].

Existing antenatal, delivery and postpartum care interventions, are proven to improve maternal and newborn outcomes [25] and reduction in maternal and perinatal mortality requires the appropriate, sustainable provision of these services to those who need them most [26]. Examples include immunization for tetanus, and prenatal iron-folate supplementation; these antenatal interventions have shown clear survival benefits for both mother and baby [26]. Registry estimates for each of these factors are consistent with PDHS 2012-13 estimates for Sindh province and represent a significant improvement over PDHS 2006-07. However, the wide differences between the Pakistan and the other sites in uptake of antenatal interventions such as iron-folate supplementation and immunization for tetanus, are likely to be contributing factors for the poor maternal and newborn outcomes in Pakistan.

Delivery complications are responsible for half of all maternal deaths, one-third of stillbirths and a quarter of neonatal deaths [27-29]. These complications, which are not easily predicted, usually first become apparent during labor and often require timely facility-based management to avert death and severe morbidity [30]. A lack of life-saving delivery care may be attributed to an inability to recognize an impending complication, failure to reach an appropriate level of care in a timely manner, a lack of appropriate care provision at the facility or to iatrogenic causes, for example from unsafe labor augmentation or unhygienic care practices [31]. Interestingly, the Registry data do not demonstrate a large deficit in the proportion of deliveries carried out at facilities or attended by skilled birth attendants in the Pakistan compared to the African and Latin American sites. On the other hand, the Indian sites have made enormous progress towards universal skilled attendance at the time of delivery. What is less clear is whether the quality of the facility care in the Pakistan site is equal to that in the other sites. Indicators of newborn care that should be the standard of care for facility deliveries, e.g. cord care and newborn immunization are not widespread in any of the sites. However, the Pakistan site fares particularly poorly in provision of these key life-saving interventions that may also be responsible for the high mortality rates.

There is variation in birth weight between the Pakistani and Indian sites, which may explain a portion of the poor newborn outcomes; however, the differences are not large enough to account for multiple-fold difference in perinatal and neonatal mortality rates. Furthermore, the excess mortality in the Pakistan site is largely concentrated amongst normal birth weight babies. Comparing the African and Pakistani sites, the proportion of babies born with low birth weight are much higher in the latter; however, there is actually no difference in survival of infants born with a weight below 2500 g in these sites. Neither the Pakistani, nor the two African sites have made progress on saving the lives of low birth weight newborns. However, the huge differential between maternal mortality, stillbirth rates and neonatal mortality amongst normal weight babies suggests that neither the Pakistani nor the African sites have managed to implement the higher level of care required to save preterm and or LBW babies. The African sites appear to have had more success in saving the lives of full-term or normal weight babies. In addition to this finding, the African sites have significantly fewer LBW newborns, thus improving the outcomes overall.

## Limitations

The Registry is limited to particular regions in each country and may not be representative of the country as a whole. However, sentinel surveillance sites, such as the MNHR sites, can be used to signal trends and identify changes in the burden of disease, providing a source of data in the absence of robust vital registration systems. While the HDI ranking of Thatta is in the bottom half of districts in Pakistan, the presence of health care facilities and the relatively easy access to both Karachi and Hyderabad for more advanced levels of care is better than in many places in Pakistan. Thus changes in Thatta may foreshadow national level trends and should signal the need for early evaluation of maternal and neonatal mortality in the country as a whole.

Women are enrolled in the Registry at approximately 20 weeks of gestation. Thus it is possible that some maternal deaths early in pregnancy could have been missed, although every maternal death known to the registry administrators was included in the statistics presented, even if they occurred prior to enrollment in the registry. In sites such as Pakistan where unsafe abortions are common, abortion related deaths might lead to an overall underestimation of the MMR. For the purposes of comparison, however, the methodology was consistent across the sites. In addition, enrollment during the second trimester precluded measurement of pre-pregnancy weight or weight gain during pregnancy, thus limiting the ability to assess the impact of poor nutrition, which may be a crucial factor in poor pregnancy outcomes in Pakistan. We also did not have data on caloric or vitamin intake during pregnancy as collection of these types of data was not practical for a large registry study.

Some factors that have been found to be associated with poor maternal and newborn outcomes are not included in this paper. For example, the Registry at the Global Network sites collect data on anemia from women who have had a determination of hemoglobin level during their routine antenatal care. However, in Pakistan routine measurement of hemoglobin is rare, thus comparable information regarding anemia was not available for analysis. Anemia is a significant factor contributing to maternal mortality and is ubiquitous amongst low-income women in Pakistan [32], thus failure to capture this information undermines the ability to construct a comprehensive explanatory model.

Even for variables that were captured and analyzed, e.g. for antenatal iron/vitamins, a lack of detail regarding formulation and frequency of intake limited the ability to assess the effectiveness of the interventions provided. This was particularly true for emergency obstetric and newborn (EmONC) interventions that are considered crucial for saving lives in the peripartum period. Only limited data regarding the need, availability, implementation or effectiveness of EmONC interventions in individual cases was present.

Additionally, these data were unable to account for macro-level differences between the sites, e.g. in Pakistan the health system is dominated by private providers in a fee for service model compared to the Indian sites where the public sector is the major provider of services. Furthermore, unprecedented floods affected the Pakistani site during the monsoon seasons of 2010 and 2011. The impact of these natural disasters on both the population and the health care system could not be captured. Nonetheless, the effect of the floods in Thatta district closely mirrored that in the rest of the country. Approximately 10% of the population of the district was affected, which is similar to the proportion of the total population of the country that was affected.

## Conclusion

The MNHR of the Global Network is the largest prospective, community-based study to evaluate maternal mortality and it demonstrates that the Pakistani site of the Global Network has significantly worse maternal and newborn health outcomes than the Network’s other six sites across five countries with similar economic and developmental indicators. Particularly concerning is the increase in maternal mortality in the period from 2010 to 2013. This trend would indicate that gains in maternal, fetal and neonatal mortality made in Pakistan between 1990 and 2006 are being reversed and occurs in the face of apparent improvement in some MNH process indicators. Despite these improvements, the increase in maternal mortality and the stagnation of feto-neonatal outcomes indicates that currently implemented programs for improving MNH in Pakistan are unlikely to bring about the desired outcomes.

As evidenced by the comparison with the other Network sites, the current levels of antenatal and newborn care interventions, while representing an increase for the Pakistani site, are insufficient to protect against poor maternal and neonatal outcomes. On the other hand, delivery care in the Pakistani site, whilst numerically equivalent to sites in Africa, is apparently ineffective in saving the lives of delivering women and their fetuses/newborns. There is an urgent need to understand the gaps that exist in facility/skilled delivery care. It is likely that the quality of delivery care in Pakistan is poor. If significant progress is to be made in saving maternal, fetal and neonatal lives, then business-as-usual in the existing health system will not just prevent Pakistan from achieving the Millennium Development Goals but may lead to a reversal of gains made over the two decades from 1990-2010.

## Competing interests

The authors declare that they have no competing interests.

## Peer review

Reviewer reports for this article can be found in Additional file 1.

## Supplementary Material

Additional file1 Click here for file

## Declarations

This article has been published as part of *Reproductive Health* Volume 12 Supplement 2, 2015: Research reports from the NICHD Global Network for Women’s and Children’s Health Research Maternal and Newborn Health Registry. The full contents of the supplement are available online at http://www.reproductive-health-journal.com/supplements/12/S2. Publication of this supplement was supported by grants from the Eunice Kennedy Shriver National Institute of Child Health and Human Development to RTI International.
