# Supplementary material for: Maternal and newborn outcomes in Pakistan compared to other low and middle income countries in the Global Network’s Maternal Newborn Health Registry: an active, community-based, pregnancy surveillance mechanism
Source: Reprod Health. 2015 Jun 8;12(Suppl 2):S15. doi: 10.1186/1742-4755-12-S2-S15 (PMC4464035; doi:10.1186/1742-4755-12-S2-S15)
Supplement: Additional file1 [file 1742-4755-12-S2-S15-S1.pdf]

Referee's comments to the authors– this sheet WILL be seen by the author(s) and published with the article

Title	Diverging maternal, fetal and neonatal outcomes: Pakistan and other low and middle income countries in the Global Network's Maternal Newborn Health Registry
Author(s)	Omrana Pasha, Sarah Saleem, Sumera Ali, Shivaprasad S Goudar, Ana Garces, Fabian Esamai, Archana Patel, Elwyn Chomba, Fernando Althabe, Janet Moore, Margo Harrison, Mabel B Berrueta, K Michael Hambidge, Nancy F Krebs, Patricia L Hibberd, Waldemar A Carlo, Bhala Kodkany, Richard J Derman, Edward A Liechty, Marion Koso-Thomas, Elizabeth M McClure, and Robert L Goldenberg
Referee's name	Stephen Hodgins

When assessing the work, please consider the following points, where applicable:

1. Is the question posed by the authors new and well defined?
2. Are the methods appropriate and well described, and are sufficient details provided to replicate the work?
3. Are the data sound and well controlled?
4. Does the manuscript adhere to the relevant standards for reporting and data deposition?
5. Are the discussion and conclusions well balanced and adequately supported by the data?
6. Do the title and abstract accurately convey what has been found?
7. Is the writing acceptable?

Please make your report as constructive and detailed as possible in your comments so that authors have the opportunity to overcome any serious deficiencies that you find and please also divide your comments into the following categories:

- Major Compulsory Revisions (which the author must respond to before a decision on publication can be reached)
- Minor Essential Revisions (such as missing labels on figures, or the wrong use of a term, which the author can be trusted to correct)
- Discretionary Revisions (which are recommendations for improvement but which the author can choose to ignore)

Where possible please supply references to substantiate your comments.

When referring to the manuscript please provide specific page and paragraph citations where appropriate.

General comments: I think it's a good, sound paper, for the most part.

Major compulsory revisions:

Minor essential revisions: There are a number of places in the paper where the language used over-generalizes from study site to the whole country (see more specific comments in attached commented pdf). Where the authors want to make a point that generalizes to the country, this needs to be adequately supported with data from other sources.

Discretionary revisions:

(continue on the next sheet)

1. **Is the question posed by the authors new and well defined?**

The question is well-defined. It not necessarily highly novel. But the data from their study site (compared with equivalent sites elsewhere) offers useful contrasts, which help draw attention to important drivers of the observed worse outcomes in the Pakistan site.

2. **Are the methods appropriate and well described, and are sufficient details provided to replicate the work?**

Yes

3. **Are the data sound and well controlled?**

Yes, they appear to be. However, the authors need to be more cautious in over-generalizing to Pakistan as a whole. For example, while they may have demonstrated a statistically significant increase in MMR in their study area, one cannot therefore conclude that there has been a concomitant increase nationwide.

4. Does the manuscript adhere to the relevant standards for reporting and data deposition?

Yes

5. Are the discussion and conclusions well balanced and adequately supported by the data?

Yes (though see my comments on over-generalization)

6. Do the title and abstract accurately convey what has been found?

See comments in abstract in attached pdf

7. Is the writing acceptable?

yes

:

Referee's comments to the authors– this sheet WILL be seen by the author(s) and published with the article

Title	Diverging maternal, fetal and neonatal outcomes: Pakistan and other low and middle income countries in the Global Network's Maternal Newborn Health Registry
Author(s)	Omrana Pasha, Sarah Saleem, Sumera Ali, Shivaprasad S Goudar, Ana Garces, Fabian Esamai, Archana Patel, Elwyn Chomba, Fernando Althabe, Janet Moore, Margo Harrison, Mabel B Berrueta, K Michael Hambidge, Nancy F Krebs, Patricia L Hibberd, Waldemar A Carlo, Bhala Kodkany, Richard J Derman, Edward A Liechty, Marion Koso-Thomas, Elizabeth M McClure, and Robert L Goldenberg
Referee's name	Yasmin Neggers

When assessing the work, please consider the following points, where applicable:

1. **Is the question posed by the authors new and well defined?**
2. **Are the methods appropriate and well described, and are sufficient details provided to replicate the work?**
3. **Are the data sound and well controlled?**
4. **Does the manuscript adhere to the relevant standards for reporting and data deposition?**
5. **Are the discussion and conclusions well balanced and adequately supported by the data?**
6. **Do the title and abstract accurately convey what has been found?**
7. **Is the writing acceptable?**

Please make your report as constructive and detailed as possible in your comments so that authors have the opportunity to overcome any serious deficiencies that you find and please also divide your comments into the following categories:

- Major Compulsory Revisions (which the author must respond to before a decision on publication can be reached)
- Minor Essential Revisions (such as missing labels on figures, or the wrong use of a term, which the author can be trusted to correct)
- Discretionary Revisions (which are recommendations for improvement but which the author can choose to ignore)

Where possible please supply references to substantiate your comments.

When referring to the manuscript please provide specific page and paragraph citations where appropriate.

General comments:

This is a well written retrospective follow up study of secondary data analysis of extensive data about maternal and child outcomes collected from several continents. But, it suffers from some major flaws, which if not corrected make the results not valid and unpublishable.

Major compulsory revisions:

1. The Pakistani sites used in this manuscript which supply the back bone of the conclusions drawn in the study, were affected by unprecedented floods during the monsoon of 2010 and 20111 (page 12, second paragraph). As the authors themselves indicate, this disrupted the health care system. Also, it is obvious that food supply to the pregnant women at Pakistani sites will be severely affected. Please use data from other sites in Pakistan which were not affected during the data collection for this manuscript.
2. Nowhere in the study is food intake or Calorie intake of pre-pregnant or pregnant women mentioned. Without some estimate of macro or energy nutrients the neonatal and maternal results from various countries cannot be compared. Just the mention of similar socio-economic status is not sufficient.
3. Study has reported that only maternal parity and education were associated with maternal and neonatal mortality. Authors need to report the proportion of variance in maternal mortality explained by these two variables. What was the R^2 for the model used to analyze this data? Was weight gain during pregnancy recorded? I understand that this was a retrospective follow up study, but without taking into account certain critical factors related to outcomes of neonatal and maternal mortality, the results of only maternal parity and education as predictors of outcomes is not valid.
4. It is hard to believe that pre-pregnancy weight, a universally accepted factor, was not associated with neonatal mortality. Was weight gain during pregnancy reported? Need to take this into account, because it reflect energy intake of pregnant women.
5. Authors are concerned that iron and other micronutrient intakes are not accounted for. But surely, without enough energy, these nutrients will not be very effective.

6. I would suggest that data from sites unaffected by floods in Pakistan be analysed for this manuscript.

7. If no data on energy intake and weight gain for the study population is available, then these should be discussed as serious weaknesses of the study.

8. It is not clear from the reference provided for methodology in this manuscript how the data analysis was conducted. Please provide results of multiple regression analysis for each country using various known confounders for neonatal and maternal mortality along with maternal education and parity. Report the R^2 of each model.

Continued:
All my comments are of major concern and should be dealt with carefully by the authors before acceptance of this manuscript.

Supplement Editor comments:

As you can see the reviewers raised serious concerns about your manuscript. Please careful address all of them and your replies should be reflected in changes in the manuscript.
